# Distinct mortality patterns and sudden cardiac death mechanisms in heart failure with a preserved ejection fraction

**DOI:** 10.1038/s41598-025-20924-8

**Published:** 2025-10-23

**Authors:** Yoshihiro Sobue, Eiichi Watanabe, Masanobu Yanase, Hideo Izawa

**Affiliations:** 1 Department of Cardiology, Fujita Health University Bantane Hospital, 3-6-10 Otobashi, Nakagawa-Ku, Nagoya, Aichi 454-8509 Japan; 2 Department of Cardiology, Fujita Health University School of Medicine, Toyoake, Japan

**Keywords:** Sudden cardiac death, HFpEF, QTc, Ventricular arrhythmias, Risk stratification, Cardiology, Cardiovascular biology

## Abstract

Sudden cardiac death (SCD) is a leading cause of mortality in heart failure (HF), yet its incidence, mechanisms, and predictors in heart failure with preserved ejection fraction (HFpEF) remain poorly characterized. We conducted a prospective study of 2331 patients hospitalized for decompensated HF, stratifying them into HFpEF (n = 754), HF with mid-range ejection fraction (HFmrEF, n = 369), and HF with reduced ejection fraction (HFrEF, n = 1208). Over a median 25-month follow-up, 298 patients (12.8%) experienced SCD. HFpEF patients had the lowest SCD incidence (5.9%) compared to HFmrEF (13.8%) and HFrEF (16.7%) (*p* < 0.01). Asystole predominated in HFpEF SCD cases (52.9%), whereas ventricular tachyarrhythmias dominated in HFmrEF and HFrEF. In multivariate competing risk analysis, NYHA class III (cause-specific hazard ratio [csHR] 2.04, 95% CI 1.20–3.45, *p* < 0.01; subdistribution hazard ratio [sHR] 1.56, 95% CI 1.16–1.96, *p* = 0.01) and prolonged QTc interval > 480 ms (csHR 1.63, 95% CI 1.03–2.55, *p* = 0.01; sHR 1.57, 95% CI 1.04–2.38, *p* = 0.03) independently predicted SCD in patients with HFpEF. These findings reveal distinct SCD patterns in HFpEF and underscore the need for phenotype-specific risk stratification and prevention strategies.

**Supplementary Information:**

The online version contains supplementary material available at 10.1038/s41598-025-20924-8.

## Introduction

Heart failure (HF) is a leading global cause of morbidity and mortality, with sudden cardiac death (SCD) accounting for a significant proportion of deaths among patients with HF^1^. Traditionally, research on SCD in HF patients has primarily focused on those with a reduced ejection fraction (HFrEF), given the well-established benefits of implantable cardioverter defibrillators (ICDs) in this population^2^. However, patients with a preserved ejection fraction (HFpEF) now constitute an increasing proportion of the HF population due to aging demographics and the growing prevalence of comorbidities such as hypertension, diabetes, and obesity^3^.

Despite the increasing prevalence of HFpEF, the risk and mechanisms of SCD in this population remain poorly understood, creating a significant gap in our ability to develop targeted interventions^4^. Unlike HFrEF, where the SCD risk is predominantly associated with arrhythmic events, the etiology of SCD in HFpEF appears to be more heterogeneous, including non-arrhythmic causes such as acute decompensated HF^5^. Furthermore, prognostic tools for SCD risk stratification in HFpEF are limited, leaving a substantial gap in the ability to guide preventive strategies in this population.

Emerging evidence suggests that the risk of SCD varies significantly across the spectrum of HF phenotypes, with intermediate ejection fraction (HFmrEF) patients showing characteristics overlapping with both HFrEF and HFpEF^6^. Large-scale studies focusing on detailed phenotyping and longitudinal outcomes are essential to delineate these differences and identify the patients at the highest risk of SCD^7^.

Therefore, this study analyzed SCD incidence across HF phenotypes and identified prognostic factors in HFpEF to enhance risk stratification and develop precision management strategies.

## Methods

### Enrollment

**Fig. 1:**
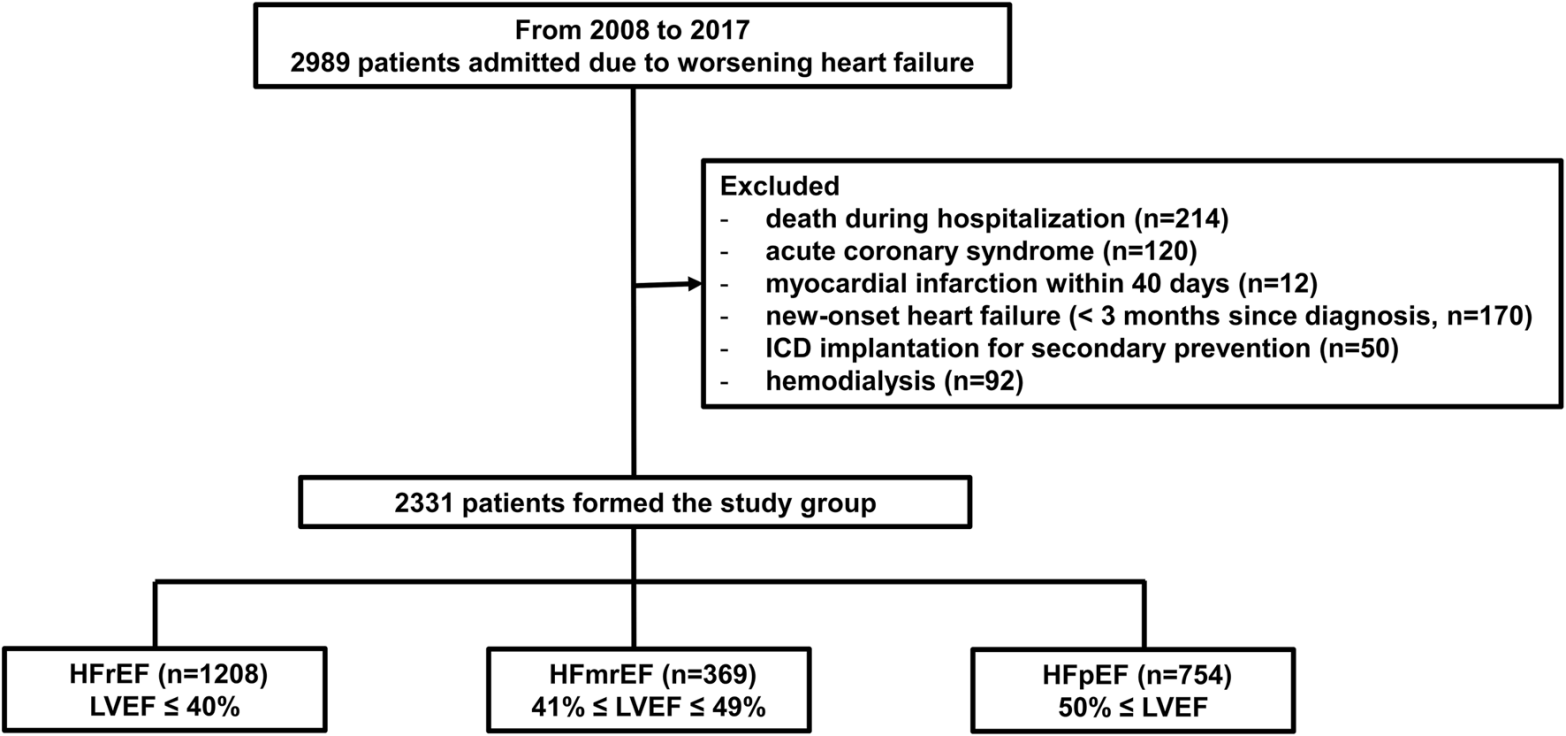
Study Population Flowchart. From an initial cohort of 2989 patients admitted for worsening heart failure between 2008 and 2017, 2331 were categorized into three phenotypes based on the left ventricular ejection fraction: HFrEF (n = 1208), HFmrEF (n = 369), and HFpEF (n = 754).

Between January 2008 and December 2017, a total of 2989 consecutive patients hospitalized at our institution for decompensated HF were prospectively enrolled in this study (Fig. 1). Upon admission, all patients underwent a comprehensive clinical evaluation, which included a detailed medical history, physical examination, measurement of vital signs, laboratory assessments, 12-lead electrocardiogram, and transthoracic echocardiogram. For analytical purposes, these data were collected and recorded at the time of hospital discharge.

The diagnosis of HF required the presence of at least one symptom (e.g., dyspnea, orthopnea, or peripheral edema) and one clinical sign (e.g., pulmonary rales, ascites, peripheral edema, or radiographic evidence of pulmonary vascular congestion)^8^. The HF types were categorized using the left ventricular ejection fraction (LVEF) as follows: HFpEF (≥ 50%), HFmrEF (41–49%), and HFrEF (< 40%)^9^. The diagnostic criteria for HFpEF were based on established guidelines^8,9^, and included clinical symptoms, LVEF ≥ 50%, natriuretic peptide elevation, and echocardiographic evidence of diastolic dysfunction: (1) clinical signs and symptoms consistent with HF, (2) LVEF ≥ 50%, (3) elevated B-type Natriuretic Peptide (BNP) levels (> 80 pg/mL in sinus rhythm or > 240 pg/mL in atrial fibrillation), and (4) echocardiographic evidence of diastolic dysfunction, including an average septal-lateral E/e' ratio > 15 and a relative wall thickness > 0.42^10^. To evaluate the concordance with contemporary diagnostic frameworks, we also calculated the HFA-PEFF and H2FPEF scores retrospectively using discharge data. These scores were used for descriptive comparison and exploratory prognostic analysis^11,12^.

Patients were excluded from the study if they met any of the following criteria: in-hospital death, New York Heart Association (NYHA) functional class IV at discharge, newly diagnosed HF within the past three months, acute coronary syndrome within the preceding 40 days, recent coronary revascularization within 90 days, prior implantation of an ICD or cardiac resynchronization therapy defibrillator for secondary prevention, or end-stage kidney disease requiring dialysis. Additionally, patients with a clinical diagnosis of hypertrophic cardiomyopathy or cardiac amyloidosis, confirmed by imaging or histologic evaluation, were excluded from the analysis.

This study was conducted in compliance with the Declaration of Helsinki and was approved by the Institutional Review Board of Fujita Health University School of Medicine (HM20-161). Written informed consent was obtained from all participants. Event classifications and patient data were independently reviewed by two cardiologists, with a third cardiologist consulted in cases of disagreement to ensure consistency.

### Follow-up and documentation of the endpoints

For patients who continued follow-up care at our institution, their clinical status was assessed using detailed reviews of their medical records. For those who received follow-up outside our hospital, information was collected through structured telephone interviews conducted with either the patient’s family or their primary care physician.

The primary outcome of this study was SCD, defined as an unexpected, non-traumatic fatal event occurring within 24 h of symptom onset^13^. That included cases associated with documented ventricular tachycardia (VT) or ventricular fibrillation (VF), as well as instances of appropriate ICD therapy delivered for VT or VF^14^. The appropriateness of ICD therapies was independently evaluated and confirmed by two experienced electrophysiologists to ensure an accurate classification^15^.

### Statistical analyses

Continuous variables were presented as the mean ± standard deviation (SD) or as medians with interquartile ranges (25th and 75th percentiles). Comparisons between groups were conducted using a Student’s t-test for normally distributed data. Categorical variables were described as frequencies and analyzed using either the χ^2^ test or Wilcoxon signed-rank test, depending on the data distribution. Continuous variables that demonstrated statistical significance (*p* < 0.05) in the univariate analysis were further divided into quartiles for subgroup analyses.

To evaluate the differences across multiple groups, a Kruskal–Wallis one-way analysis of variance was employed, followed by Tukey’s post-hoc tests to identify specific group differences. To appropriately address the presence of competing events, we estimated cumulative incidence functions (CIFs) for SCD, explicitly treating non-SCD death as a competing risk. Comparisons of CIFs across groups were performed using Gray’s test. For prognostic modeling, we applied both cause-specific Cox proportional hazards models—which estimate the instantaneous risk of SCD while censoring non-SCD deaths—and Fine–Gray subdistribution hazard models, which directly quantify the effect of covariates on the cumulative incidence of SCD in the presence of competing events. Results are expressed as cause-specific hazard ratios (csHRs) and subdistribution hazard ratios (sHRs), each with corresponding 95% confidence intervals. All statistical analyses were conducted using JMP17 software (SAS Institute, Cary, NC, USA) and R software (version 4.5.1; R Foundation for Statistical Computing, Vienna, Austria).

## Results

### Patient characteristics

The baseline characteristics of the enrolled cohort are detailed in Table 1. The mean age of the participants was 74 ± 13 years, with 57% being male. The average left ventricular ejection fraction (LVEF) was 40 ± 15%. At the time of discharge, most patients (87%) were classified as New York Heart Association (NYHA) functional class II.

**Table 1:** Patient Characteristics according to type of heart failure.

	HFrEF (n = 1208)	HFmrEF (n = 369)	HFpEF (n = 754)	*P*-value
Age (years)	72 ± 14	76 ± 12	76 ± 12	< 0.01
Male sex – no. (%)	779 (64)	202 (55)	344 (46)	< 0.01
*Co-morbidities – no. (%)*				
Hypertension	820 (68)	279 (76)	578 (77)	< 0.01
Diabetes mellitus	481 (40)	169 (46)	247 (33)	< 0.01
Dyslipidemia	478 (40)	154 (42)	259 (34)	0.02
Ischemic heart disease	707 (59)	191 (52)	232 (31)	< 0.01
Atrial fibrillation	382 (32)	146 (40)	311 (41)	< 0.01
NYHA functional class – no. (%)				0.43
II	1038 (86)	328 (89)	664 (88)	
III	170 (14)	41 (11)	90 (12)	
*Blood pressure – mmHg*				
Systolic	108 ± 36	112 ± 24	114 ± 39	< 0.01
Diastolic	61 ± 16	63 ± 32	61 ± 16	0.33
Heart rate – beats/min	72 ± 19	71 ± 17	73 ± 31	0.31
*Laboratory data*				
Hemoglobin – g/dl	12.1 ± 2.3	11.3 ± 2.4	11.0 ± 2.3	< 0.01
BUN – mg/dl	27.6 ± 17.4	30.7 ± 29.2	27.6 ± 22.5	0.07
Serum creatinine – mg/dl	1.5 ± 1.5	1.8 ± 2.1	1.4 ± 1.5	< 0.01
eGFR (mL/min/1.73 m^2^)	48.6 ± 26.4	46.0 ± 28.2	49.5 ± 25.6	0.11
Sodium – mEq/L	139 ± 5	139 ± 5	139 ± 5	0.60
Potassium – mEq/L	4.1 ± 0.7	4.2 ± 0.7	4.1 ± 0.7	0.11
BNP – pg/ml	285 (145–587)	268 (126–524)	178 (86–364)	< 0.01
*Electrocardiography*				
QRS interval – ms	117 ± 30	111 ± 29	105 ± 25	< 0.01
QRS ≥ 120 ms – no. (%)	423 (35)	96 (26)	164 (22)	< 0.01
LBBB	139 (12)	17 (4.6)	20 (3)	< 0.01
RBBB	94 (8)	37 (10)	75 (10)	0.22
IVCD	99 (8)	24 (6.5)	19 (3)	< 0.01
Paced	87 (7)	20 (5)	50 (7)	0.87
QT interval – ms	389 ± 64	384 ± 61	398 ± 67	< 0.01
QTc interval – ms	468 ± 44	457 ± 52	450 ± 52	< 0.01
*Echocardiography*				
LVEF – %	27 ± 7	44 ± 3	58 ± 5	< 0.01
LAD – mm	41 ± 8	40 ± 8	41 ± 9	0.24
Interventricular septum – mm	10 ± 2	11 ± 3	11 ± 2	< 0.01
Posterior wall—mm	10 ± 2	11 ± 2	11 ± 2	< 0.01
E/e’	22 ± 12	21 ± 17	21 ± 25	0.84
*Medications – no. (%)*				
Beta-blocker	895 (74)	248 (67)	405 (54)	< 0.01
RAS antagonist	760 (63)	226 (61)	453 (60)	0.44
Diuretic	936 (77)	265 (72)	517 (69)	< 0.01
MRA	629 (52)	151 (41)	292 (39)	< 0.01
Statin	488 (40)	154 (42)	239 (32)	< 0.01
Antiplatelet	564 (42)	148 (40)	216 (29)	< 0.01
Oral anticoagulant	347 (29)	112 (30)	253 (34)	0.08
Digitalis	105 (9)	27 (7)	59 (8)	0.63

SCD, sudden cardiac death; NYHA, New York Heart Association; BUN, blood urea nitrogen; eGFR, estimated glomerular filtration rate; AST, aspartate transaminase; ALT, alanine aminotransferase; BNP, B-type natriuretic peptide; LBBB, left bundle branch block; RBBB, right bundle branch block; IVCD, intraventricular conduction delay; LVEF, left ventricular ejection fraction; LAD, left atrial diameter; PA; pulmonary artery; RAS, renin-angiotensin system; MRA, mineralocorticoid-receptor antagonists. Data are presented as the mean ± SD or number (%) or medians (25^th^ and 75^th^ percentile).

During the median follow-up of 25 months, 47 patients with HFrEF received either an ICD (n = 18) or cardiac resynchronization therapy device with a defibrillator (n = 29) as primary prevention of SCD. The study population was categorized into three groups based on the LVEF: HFrEF (n = 1208), HFmrEF (n = 369), and HFpEF (n = 754). Significant differences were observed among these groups in terms of the age and sex distribution (*p* < 0.01). The prevalence of comorbid conditions, including hypertension, diabetes mellitus, dyslipidemia, ischemic heart disease, and atrial fibrillation, varied significantly among the groups (*p* < 0.01 for all except dyslipidemia, *p* = 0.02).

There were no significant differences in the proportion of patients with each NYHA functional class across the groups (*p* = 0.43). However, the BNP levels and electrocardiographic parameters, including the QRS duration and prevalence of a prolonged QRS, demonstrated significant intergroup differences (*p* < 0.01). Echocardiographic measures such as the LVEF, left atrial diameter, interventricular septum thickness, and posterior wall thickness also exhibited notable variations across the groups (*p* < 0.01 for all).

Regarding medication use, beta-blockers, mineralocorticoid receptor antagonists (MRAs), and diuretics were more frequently prescribed for patients with HFrEF compared to those with HFmrEF or HFpEF (*p* < 0.01 for all). In contrast, the use of renin-angiotensin system (RAS) antagonists and oral anticoagulants did not significantly differ among the groups (*p* = 0.44 and *p* = 0.08, respectively).

### Outcomes

**Fig. 2:**
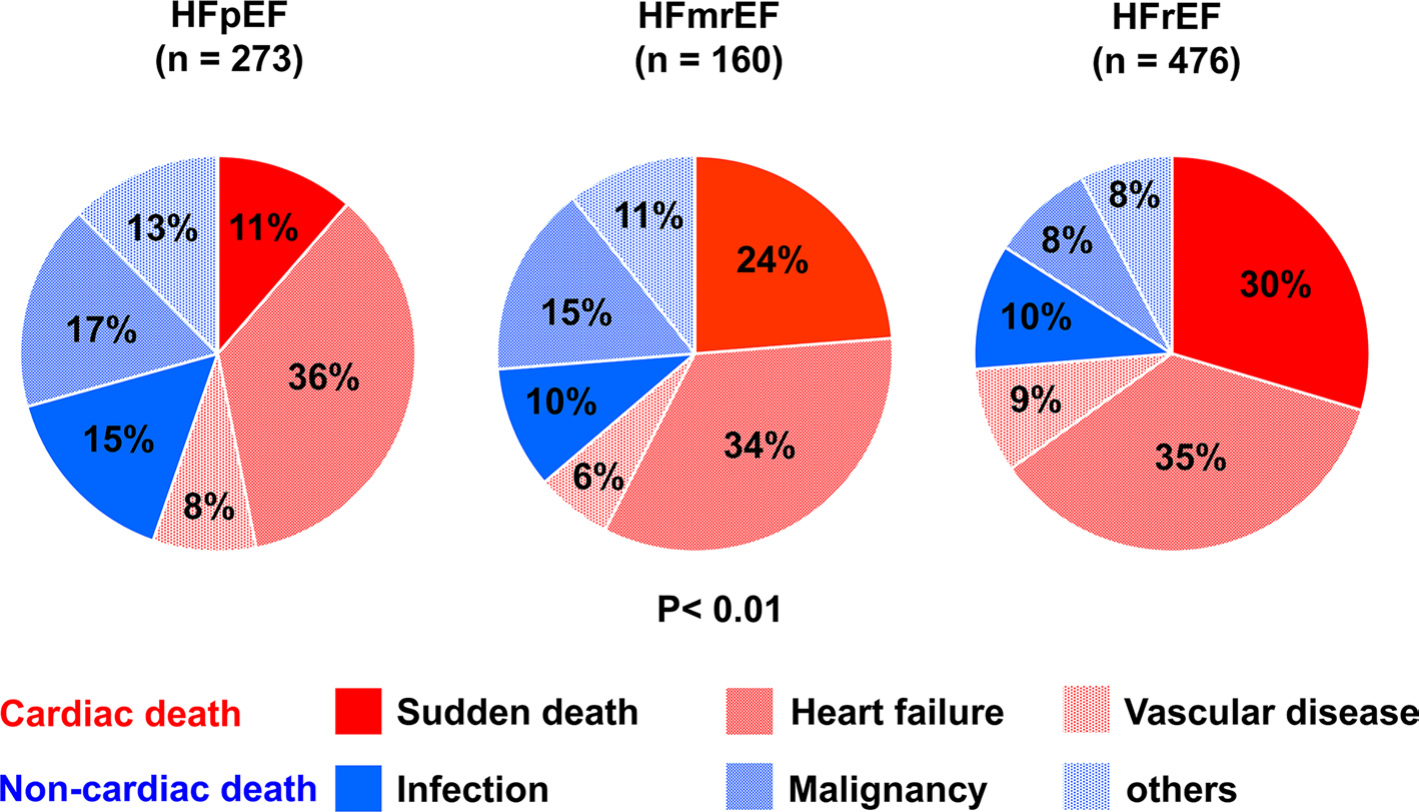
Distribution of the Causes of Death Across the Heart Failure Phenotypes. The proportions of cardiac and non-cardiac deaths are displayed for the patients with HFrEF, HFmrEF, and HFpEF. Significant differences were observed in the distribution, with cardiac deaths being more prevalent in HFrEF and HFmrEF, whereas non-cardiac deaths were dominant in HFpEF (*p* < 0.01).

**Fig. 3:**
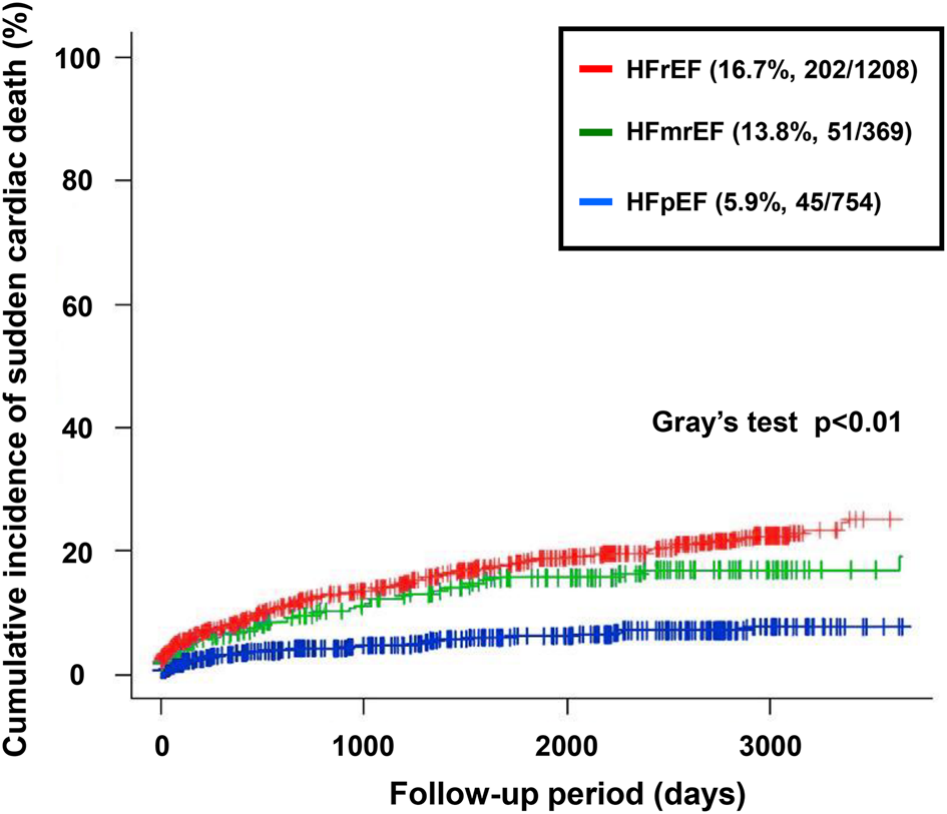
Cumulative Incidence Functions for Sudden Cardiac Death. Cumulative incidence functions for sudden cardiac death (SCD), stratified by HF phenotype, with non-SCD death treated as a competing event. Between-group differences were assessed using Gray’s test (*p* < 0.01).

During the median follow-up period of 25 months, 909 (38.9%) patients died. Among those, 298 (12.8%) patients developed SCD. Among these, 285 cases (95.6%) of SCD were confirmed upon emergency presentation to our hospital, while 13 cases (4.3%) were identified through telephone interviews with the patients’ families. The distribution of causes of death varied significantly according to the heart failure phenotypes. SCD was less frequent in patients with HFpEF (11%) compared to HFrEF (36%) and HFmrEF (24%) (*p* < 0.01). In contrast, non-cardiac deaths were more prevalent in HFpEF patients (45%) than in those with HFrEF (26%) or HFmrEF (36%) (*p* < 0.01) (Fig. 2). The CIFs for SCD across HF phenotypes are presented in Fig. 3. HFpEF patients exhibited the lowest incidence of SCD (5.9%, 45/754), while HFmrEF and HFrEF patients demonstrated higher incidences of 13.8% (51/369) and 16.7% (202/1208), respectively (Gray’s test *p* < 0.01).

**Fig. 4:**
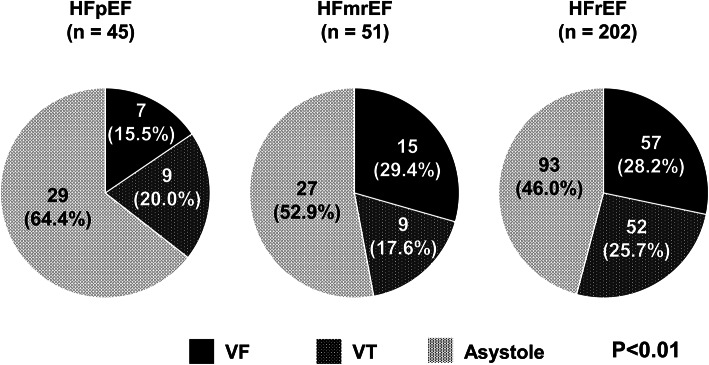
Modes of Sudden Cardiac Death by the Heart Failure Phenotype. The modes of SCD (asystole, ventricular arrhythmias) are shown for HFrEF, HFmrEF, and HFpEF. Asystole was most common in HFpEF (52.9%), while ventricular arrhythmias were predominant in HFrEF and HFmrEF (*p* < 0.01). Those findings highlight the distinct arrhythmic profiles among the heart failure phenotypes.

The mode of SCD differed significantly among the HF phonotypes (Fig. 4). In HFpEF, asystole was the most frequent mode of SCD, accounting for 52.9% of cases, compared to 17.6% in HFmrEF and 29.4% in HFrEF (*p* < 0.01). Conversely, ventricular arrhythmias, including VT and VF, were more common in HFrEF (46.0%) and HFmrEF (64.4%) than in HFpEF (28.2%) (*p* < 0.01). In addition, 47% of SCDs in HFpEF were categorized as sudden unexplained death, whereas VT/VF-related death accounted for the majority in HFrEF (68%).

### Sudden cardiac death in HFpEF

Among 754 patients with HFpEF, 45 (5.9%) experienced SCD during a median follow-up period (Table 2). In the HFpEF cohort, the mean HFA-PEFF score was 5.7 ± 1.2, with 83% of patients having a score ≥ 5. The mean H2FPEF score was 4.1 ± 0.9, with 78% of patients having a score ≥ 4. Patients with SCD were significantly younger than survivors (72 ± 16 vs. 77 ± 12 years, *p* = 0.05) and were more frequently male (67% vs. 44%, *p* < 0.01). The prevalence of atrial fibrillation was lower among the SCD patients than the survivors (23% vs. 42%, *p* = 0.02).

**Table 2:** Baseline characteristics in patients with HFpEF.

	Overall (n = 754)	Survivors (n = 709)	SCD (n = 45)	*P*-value
Age (years)	76 ± 12	77 ± 12	72 ± 16	0.05
Male sex – no. (%)	344 (46)	314 (44)	30 (67)	< 0.01
*Co-morbidities – no. (%)*				
Hypertension	578 (77)	545 (77)	33 (74)	0.71
Diabetes mellitus	247 (33)	230 (32)	17 (37)	0.62
Dyslipidemia	259 (34)	244 (34)	15 (33)	0.87
Ischemic heart disease	232 (31)	216 (30)	16 (35)	0.49
Atrial fibrillation	311 (41)	301 (42)	10 (23)	0.02
NYHA functional class at discharge – no. (%)				0.04
II	664 (88)	628 (89)	36 (79)	
III	90 (12)	81 (11)	9 (21)	
*Blood pressure at discharge – mmHg*				
Systolic	114 ± 39	115 ± 40	107 ± 31	0.12
Diastolic	61 ± 16	61 ± 14	60 ± 17	0.70
Heart rate at discharge– beats/min	73 ± 31	73 ± 31	70 ± 16	0.30
*Laboratory data at discharge*				
Hemoglobin – g/dl	11.0 ± 2.3	11.1 ± 2.2	10.7 ± 1.8	0.64
Platelet- × 10^4^/μL	19.2 ± 7.9	19.1 ± 7.8	18.7 ± 7.5	0.71
Albumin – g/dl	3.4 ± 0.6	3.4 ± 0.6	3.4 ± 0.7	0.82
BUN – mg/dl	28 ± 23	28 ± 20	31 ± 21	0.36
Serum creatinine – mg/dl	1.4 ± 1.5	1.4 ± 1.5	1.7 ± 1.5	0.29
eGFR – mL/min/1.73 m^2^	50 ± 26	51 ± 26	49 ± 28	0.62
Sodium – mEq/L	139 ± 5	139 ± 8	137 ± 5	0.05
Potassium – mEq/L	4.1 ± 0.7	4.1 ± 0.7	4.3 ± 0.7	0.27
AST – IU/L	39 ± 11	40 ± 11	27 ± 13	0.08
ALT – IU/L	27 ± 13	27 ± 12	22 ± 14	0.08
BNP – pg/ml	178 (86 – 364)	168 (86 – 366)	290 (98 – 406)	0.92
*Electrocardiography*				
QRS duration – ms	105 ± 25	105 ± 27	111 ± 34	0.29
QRS ≥ 120 ms – no. (%)	164 (25)	154 (22)	10 (23)	0.68
LBBB	20 (3)	18 (3)	2 (5)	0.81
RBBB	75 (12)	73 (10)	2 (5)	0.43
IVCD	19 (2)	17 (2)	2 (4)	0.20
Paced	50 (7)	46 (6)	4 (9)	0.57
QT – ms	398 ± 67	388 ± 62	404 ± 62	< 0.01
QTc – ms	450 ± 52	446 ± 51	466 ± 49	< 0.01
*Echocardiography*				
LVEF – %	58 ± 5	58 ± 11	53 ± 12	0.83
LAD – mm	41 ± 9	41 ± 10	46 ± 9	< 0.01
E/e’	21 ± 25	22 ± 26	20 ± 17	0.49
Relative wall thickness	0.53 ± 0.28	0.53 ± 0.24	0.52 ± 0.16	0.78
PA systolic pressure – mmHg	38 ± 19	39 ± 19	36 ± 16	0.54
*Medication – no. (%)*				
Beta-blocker	405 (54)	378 (53)	27 (60)	0.12
RAS antagonist	453 (60)	421 (59)	32 (70)	0.81
Diuretic	517 (69)	487 (69)	30 (67)	0.18
MRA	292 (39)	279 (39)	13 (28)	0.12
Statin	239 (32)	222 (31)	17 (37)	0.51
Antiplatelet	216 (28)	201 (28)	15 (33)	0.63
Oral anticoagulant	253 (34)	243 (34)	10 (23)	0.20
Digitalis	59 (8)	57 (8)	2 (5)	0.15
Antiarrhythmic drugs	75 (10)	72 (10)	3 (7)	0.44
H_2_FPEF score	4.1 ± 0.9	4.1 ± 0.9	4.0 ± 0.8	0.43
≥ 4 – no. (%)	588 (78)	553 (78)	35 (78)	0.66
HFA-PEEP score	5.7 ± 1.2	5.7 ± 1.2	5.3 ± 1.9	0.11
≥ 5 – no. (%)	623 (83)	587 (83)	36 (80)	0.34

SCD, sudden cardiac death; NYHA, New York Heart Association; BUN, blood urea nitrogen; eGFR, estimated glomerular filtration rate; AST, aspartate transaminase; ALT, alanine aminotransferase; BNP, B-type natriuretic peptide; LBBB, left bundle branch block; RBBB, right bundle branch block; IVCD, intraventricular conduction delay; LVEF, left ventricular ejection fraction; LAD, left atrial diameter; PA; pulmonary artery; RAS, renin-angiotensin system; MRA, mineralocorticoid-receptor antagonists. Data are presented as the mean ± SD or number (%) or medians (25^th^ and 75^th^ percentile).

A significant difference was observed in the NYHA functional class at discharge, with a higher proportion of SCD patients classified as NYHA class III (21% vs. 11%, *p* = 0.04). The prevalence of hypertension, diabetes mellitus, dyslipidemia, and ischemic heart disease was similar between the groups. However, patients with SCD had lower sodium levels (137 ± 5 vs. 139 ± 8 mEq/L, *p* = 0.05) and larger left atrial diameters (46 ± 9 vs. 41 ± 10 mm, *p* < 0.01) at discharge. There were no significant differences in the use of beta-blockers, RAS, MRAs, diuretics, or oral anticoagulants between the groups. In the multivariate competing risk analysis, adjusted for age, sex, and clinically relevant variables (Supplemental Table), an NYHA functional class III (csHR 2.04, 95% CI 1.20–3.45, *p* < 0.01; sHR 1.56, 95% CI: 1.16–1.96, *p* = 0.01), lower serum sodium concentration (csHR 0.94, 95% CI 0.89–0.99, *p* = 0.04; sHR 0.96, 95% CI 0.94–1.00, *p* = 0.08), and a prolonged QTc interval (per 10-ms increase: csHR 1.04, 95% CI 1.01–1.07, *p* = 0.02; sHR 1.02, 95% CI 1.01–1.03, *p* = 0.04; > 480 ms: csHR 1.63, 95% CI 1.03–2.55, *p* = 0.01; sHR 1.57, 95% CI 1.04–2.38, p = 0.03) were identified as independent predictors of SCD in patients with HFpEF (Table 3).

**Table 3:** Multivariate Competing Risk Analyses (Cause-Specific Cox and Fine–Gray Models) for Sudden Cardiac Death in Heart Failure with Preserved Ejection Fraction (HFpEF).

Risk factor	csHR	95% CI	*P*-value	sHR	95% CI	*P*-value
Age (years)	1.02	0.99–1.05	0.27	1.03	0.99–1.07	0.13
Male sex	1.05	0.52–2.13	0.90	1.17	0.57–2.39	0.66
NYHA functional class III	2.04	1.20–3.45	< 0.01	1.56	1.16–1.96	0.01
eGFR (mL/min/1.73 m^2^)	0.98	0.97–1.00	0.06	1.00	0.99–1.00	0.88
Sodium (mEq/L)	0.94	0.89–0.99	0.04	0.96	0.94–1.00	0.08
QTc (per 10 ms increase)	1.04	1.01–1.07	0.02	1.02	1.01–1.03	0.04
< 440 ms	Reference			Reference		
440–479 ms	1.12	0.98–1.22	0.19	1.06	0.99–1.12	0.21
> 480 ms	1.63	1.03–2.55	0.01	1.57	1.04–2.38	0.03
LAD	1.01	0.97–1.06	0.48	1.02	0.98–1.06	0.33

csHR, cause-specific hazard ratio; sHR, subdistribution hazard ratio; CI, confidence interval. SCD, sudden cardiac death; NYHA, New York Heart Association; BUN, blood urea nitrogen; eGFR, estimated glomerular filtration rate; AST, aspartate transaminase; ALT, alanine aminotransferase; BNP, B-type natriuretic peptide; LBBB, left bundle branch block; RBBB, right bundle branch block; IVCD, intraventricular conduction delay; LVEF, left ventricular ejection fraction; LAD, left atrial diameter; PA; pulmonary artery; RAS, renin-angiotensin system; MRA, mineralocorticoid-receptor antagonists. Data are presented as the mean ± SD or number (%) or medians (25^th^ and 75^th^ percentile).

## Discussion

### Major findings

In this prospective observational study, we identified three major findings: (1) HFpEF patients exhibited a distinct mortality pattern with a greater prevalence of non-cardiac deaths and significantly fewer SCDs compared to HFmrEF and HFrEF, (2) asystole was the predominant mode of SCD in HFpEF, contrasting with VT and VF, which were more common in HFmrEF and HFrEF, and (3) NYHA functional class and QTc interval emerged as significant predictors of SCD in HFpEF, highlighting their potential utility in phenotype-specific risk stratification.

### Sudden cardiac death in HFpEF: differentiating the pathophysiological mechanisms

In our study, the incidence, proportion, and mechanism of SCD differed markedly between HFpEF and HFrEF, underscoring the heterogeneity of death profiles among heart failure phenotypes. While HFrEF patients exhibited a high SCD incidence (16.6%) with a predominance of ventricular tachyarrhythmias (46.0%), HFpEF patients had a lower SCD incidence (5.9%) and asystole was the leading mechanism (52.9%). This divergence suggests fundamentally different arrhythmic substrates and risk profiles, reinforcing the need for phenotype-specific preventive strategies.This lower risk of SCD in HFpEF compared to HFmrEF and HFrEF aligns with prior studies, reinforcing the notion that HFpEF has distinct pathophysiological mechanisms^3^. Notably, the predominant causes of death in HFpEF patients were non-cardiac in nature, such as malignancies and infections, whereas cardiac causes, including SCD, were more prevalent in HFmrEF and HFrEF patients. These findings underscore the distinct etiologies underlying mortality in each HF phenotype, with non-cardiac deaths accounting for 62% of the total mortality in HFpEF patients compared to 35% in HFrEF patients.

The mechanisms driving non-cardiac deaths in HFpEF are multifaceted and largely influenced by the substantial burden of comorbid conditions, including obesity, diabetes, chronic kidney disease, and pulmonary diseases^1,16^. HFpEF patients often exhibit systemic inflammation and endothelial dysfunction, both of which exacerbate the risk of non-cardiac mortality. For instance, chronic obstructive pulmonary disease and renal insufficiency are more prevalent in HFpEF than in HFrEF, contributing to the higher rates of non-cardiac death. These findings highlight the importance of comprehensive comorbidity management as a central component of HFpEF care.

Regarding cardiac deaths, the arrhythmic profiles differed significantly among the HF phenotypes. In HFpEF, asystole was the predominant mode of SCD, in stark contrast to VT and VF, which were more common in HFrEF^13,14^. This divergence suggests that the arrhythmic mechanisms in HFpEF are less amenable to traditional interventions such as ICDs, which primarily target VT and VF. Consistent with our findings, previous studies have demonstrated limited efficacy of ICDs in reducing the SCD risk in HFpEF populations, largely due to the predominance of non-arrhythmic causes of death in this phenotype. Consistent with our findings, previous studies have demonstrated the limited efficacy of ICDs in reducing SCD risk in HFpEF populations, largely due to the predominance of non-arrhythmic causes of death in this phenotype. This supports the notion that the mechanisms and predictors of SCD are fundamentally distinct between HFpEF and HFrEF^16,17^. While SCD in HFrEF is predominantly driven by ventricular tachyarrhythmias and responds well to ICD-based therapies, SCD in HFpEF is less commonly arrhythmic and presents unique risk characteristics. While asystole was the most frequently documented terminal rhythm among SCD cases in HFpEF, this likely reflects diagnostic limitations such as the absence of continuous ECG monitoring or device interrogation, and does not necessarily preclude the presence of antecedent ventricular arrhythmias. In our prior study focusing on HFrEF patients, we identified NYHA class III/IV, reduced LVEF, and renal dysfunction (eGFR < 45 mL/min/1.73 m^2^) as independent predictors of SCD using a time-varying model^18^. By contrast, in the present HFpEF-focused analysis, prolonged QTc and NYHA class III were significantly associated with SCD risk, while LVEF and eGFR were not predictive. This divergence highlights the distinct risk profiles and pathophysiological substrates underlying SCD across HF phenotypes, suggesting that comprehensive electrophysiological and functional assessments may aid in stratifying risk and guiding phenotype-specific preventive strategies. Future research should focus on developing predictive models and therapeutic strategies tailored to the unique clinical and pathophysiological features of HFpEF.

These results underscore the critical importance of personalized medicine in heart failure management^7,15^. Recognizing the heterogeneity among HF phenotypes is fundamental to designing effective prevention and treatment strategies that address the specific risks associated with each phenotype. Advancing the care of HFpEF patients will require a paradigm shift beyond traditional arrhythmia-focused approaches, emphasizing a multidisciplinary framework that incorporates both cardiac and non-cardiac factors.

### The role of the QT interval in predicting sudden cardiac death in HFpEF

In this study, we identified the QTc interval as an independent and significant predictor of SCD in patients with HFpEF. That finding underscores the critical role of electrophysiological abnormalities in the pathogenesis of SCD in HFpEF and aligns with prior evidence emphasizing the importance of the QTc interval in cardiac risk stratification.

Large-scale population-based studies have consistently demonstrated that a prolonged QT interval is associated with an increased risk of ventricular arrhythmias and SCD across diverse cardiac conditions^19^. In patients with HFpEF, a QTc interval prolongation has been identified as a significant marker of an elevated SCD risk, highlighting its utility in arrhythmic risk stratification^19,20^. Animal model studies have further validated these findings, showing that HFpEF models exhibit prolonged QT intervals and increased electrical instability, mirroring clinical observations in human populations. For instance, in a high-salt rat model of HFpEF, a QT and QTc interval prolongation were consistently observed, reflecting the electrophysiological remodeling that occurs in HFpEF and its association with an increased arrhythmic risk^20^.

The underlying mechanisms linking the QT interval prolongation to SCD in HFpEF involve complex interactions between structural and electrical remodeling^10,21^. A prolonged QT interval reflects myocardial abnormalities, including fibrosis and ionic channel dysregulation, which predispose to malignant arrhythmias. Structural remodeling, such as fibrosis and hypertrophy, creates a substrate for heterogeneous repolarization and increased dispersion of refractoriness, facilitating reentrant arrhythmias. In animal models, myocardial fibrosis and an altered ionic channel function were observed to significantly contribute to prolonged repolarization, providing mechanistic insights into the arrhythmic risk in HFpEF. Additionally, autonomic dysregulation, frequently observed in HFpEF, exacerbates the electrical instability and further increases the susceptibility to life-threatening arrhythmias.

Clinical studies have demonstrated that HFpEF patients often exhibit electrical remodeling characterized by a prolonged QT interval and increased QT dispersion—key markers of the arrhythmic risk^20,21^. These electrophysiological changes are not only indicative of underlying myocardial dysfunction but also serve as important predictors of adverse cardiovascular outcomes. Long-term cohort studies have established a direct association between QT interval abnormalities and the incidence of SCD, reinforcing the importance of incorporating a QT interval evaluation into routine clinical practice for HFpEF patients. Furthermore, findings from animal studies underscore the translational potential of preclinical research in enhancing our understanding of the arrhythmic mechanisms specific to HFpEF. Previous studies of SCD in HFpEF have reported various predictors, such as male sex, diabetes, atrial fibrillation, and renal dysfunction^22,23^. In contrast, our study revealed novel insights by analyzing a cohort of patients hospitalized for acute decompensated heart failure, identifying QTc prolongation (> 480 ms) and NYHA class III as independent predictors of SCD. Furthermore, we demonstrated that asystole—rather than ventricular tachyarrhythmias—was the predominant terminal rhythm in HFpEF. These distinctions may reflect differences in clinical setting, such as inpatient versus outpatient management, as well as variations in comorbidity burden and endpoint adjudication methods. Taken together, our findings underscore the importance of phenotype-specific approaches to risk stratification and SCD prevention in HFpEF.

Our findings have important clinical implications. First, they emphasize the need for a comprehensive electrophysiological assessment as part of the SCD risk stratification in HFpEF^7,24^. Second, targeted interventions addressing QT interval prolongation, such as optimized medical therapy, management of comorbid conditions, and modulation of autonomic tone, could mitigate the arrhythmic risk in this population. Finally, further research is warranted to elucidate the specific mechanisms by which the QT interval prolongation contributes to SCD in HFpEF and to evaluate the efficacy of therapeutic strategies aimed at reducing the incidence of SCD.

## Limitations

This study had several limitations. First, it was conducted at a single center, which may have limited the generalizability of our findings. Second, autopsy rates to confirm the cause of death were low (4.0%), which might have affected the accuracy of the SCD classification. Future multicenter studies incorporating independent validation cohorts are warranted to confirm the generalizability of our findings and establish robust, phenotype-specific risk models for SCD in HFpEF.

Additionally, over half of the SCD cases (50.6%) were categorized as asystole upon hospital arrival, leaving uncertainty about the preceding arrhythmias such as VT or VF.

Echocardiographic assessments were only performed at discharge, and subsequent changes in those parameters during follow-up could not be evaluated. Furthermore, the number of SCD events in HFpEF patients was relatively small (n = 45), which may have limited the statistical power of the subgroup analyses. Due to the limited number of SCD events in HFpEF, the multivariable model was restricted to a small number of covariates to avoid overfitting. Future large-scale, multicenter studies will be necessary to evaluate the predictive roles of additional clinical variables such as serum electrolytes, diuretic dose, comorbidities, and right-sided heart failure. We were unable to assess the impact of important therapies such as angiotensin receptor-neprilysin inhibitors and sodium-glucose cotransporter 2 inhibitors, as those medications were not widely available in Japan during the study period. Lastly, data on non-sustained VT, an important risk factor in HF, were not available for analysis.

## Conclusions

This study revealed the distinct mortality patterns and mechanisms of SCD in HFpEF, with a higher prevalence of non-cardiac deaths, fewer SCD events compared to HFmrEF and HFrEF, and asystole as the predominant SCD mode. The NYHA functional classa and QTc interval were identified as significant predictors of SCD, highlighting their potential for risk stratification.

These findings underscored the need for phenotype-specific strategies to address SCD in HFpEF and pave the way for future research on tailored risk prediction and management approaches.

## Supplementary Information

Below is the link to the electronic supplementary material.

Supplementary Material 1

## Data Availability

The deidentified participant data will be shared on request to the corresponding author. All data will be shared, as will the study protocol. Data will be available from within 6 months after publication for a period of 1 year. Data will be shared after permission has been granted by the Committees on Ethics of Fujita Health University School of Medicine. For any kind of analyses, the data will be shared as Excel files via email.
