# Supplementary material for: Distinct mortality patterns and sudden cardiac death mechanisms in heart failure with a preserved ejection fraction
Source: Sci Rep. 2025 Oct 23;15:37048. doi: 10.1038/s41598-025-20924-8 (PMC12549995; doi:10.1038/s41598-025-20924-8)
Supplement: Supplementary file 1 — Supplementary Material 1 [file 41598_2025_20924_MOESM1_ESM.docx]

**Supplemental Table. Univariate Competing Risk Analyses (Cause-Specific Cox and Fine–Gray Models) for Sudden Cardiac Death in Heart Failure with Preserved Ejection Fraction (HFpEF).**

| **Risk factor** | **csHR** | **95% CI** | **P-value** | **sHR** | **95% CI** | **P-value** |
| --- | --- | --- | --- | --- | --- | --- |
| Age | 1.02 | 0.97 – 1.07 | 0.42 | 1.02 | 0.99 – 1.04 | 0.29 |
| Male sex | 1.25 | 0.47 – 3.33 | 0.65 | 0.89 | 0.48 – 1.63 | 0.69 |
| Hypertension | 0.64 | 0.33 – 1.24 | 0.19 | 0.59 | 0.31 – 1.14 | 0.12 |
| Diabetes mellitus | 0.64 | 0.31 – 1.35 | 0.24 | 0.60 | 0.28 – 1.26 | 0.18 |
| Dyslipidemia | 1.11 | 0.58 – 2.11 | 0.76 | 1.04 | 0.55 – 1.97 | 0.91 |
| Ischemic heart disease | 0.52 | 0.15 – 1.83 | 0.31 | 1.12 | 0.60 – 2.22 | 0.67 |
| Atrial fibrillation | 0.44 | 0.14 – 1.37 | 0.16 | 1.05 | 0.57 – 1.94 | 0.88 |
| Systolic blood pressure | 0.99 | 0.98 – 1.00 | 0.11 | 1.00 | 0.98 – 1.00 | 0.10 |
| Diastolic blood pressure | 0.99 | 0.97 – 1.00 | 0.12 | 1.00 | 0.99 – 1.02 | 0.22 |
| Heart rate | 1.00 | 0.99 – 1.01 | 0.65 | 1.00 | 0.99 – 1.01 | 0.25 |
| NYHA |  |  |  |  |  |  |
| II | Reference |  |  | Reference |  |  |
| III | 5.13 | 2.80 – 9.41 | <0.01 | 1.82 | 1.12 – 2.61 | <0.01 |
| Laboratory data |  |  |  |  |  |  |
| Hb (g/dl) | 0.80 | 0.62 – 1.04 | 0.09 | 0.93 | 0.80 – 1.09 | 0.37 |
| eGFR (mL/min/1.73 m^2^) | 0.97 | 0.95 – 0.99 | 0.04 | 0.99 | 0.99 – 1.00 | 0.41 |
| Sodium (mEq/L) | 0.94 | 0.89 – 0.99 | 0.02 | 0.95 | 0.90 – 0.99 | 0.03 |
| Potassium (mEq/L) | 1.36 | 0.91 – 2.03 | 0.13 | 1.25 | 0.87 – 1.78 | 0.22 |
| BNP (pg/ml) | 1.00 | 0.99 – 1.00 | 0.29 | 1.00 | 0.99 – 1.00 | 0.66 |
| Electrocardiography |  |  |  |  |  |  |
| QRS interval (ms) | 1.01 | 0.99 – 1.03 | 0.17 | 1.01 | 0.99 – 1.02 | 0.09 |
| LBBB | 4.62 | 1.05 – 20.4 | 0.04 | 2.83 | 0.65 – 12.3 | 0.16 |
| IVCD | 3.88 | 0.51 – 29.6 | 0.19 | 2.84 | 0.76 – 10.6 | 0.12 |
| QT interval (per 10ms increase) | 1.01 | 1.00 – 1.02 | 0.02 | 1.01 | 1.00 – 1.03 | 0.04 |
| QTc interval (per 10ms increase) | 1.04 | 1.01 – 1.06 | 0.01 | 1.02 | 1.00 – 1.06 | 0.03 |
| Echocardiography |  |  |  |  |  |  |
| LVEF (%) | 0.97 | 0.88 – 1.07 | 0.55 | 0.94 | 0.89 – 1.00 | 0.05 |
| LAD (mm) | 1.06 | 1.01 – 1.11 | 0.03 | 1.00 | 0.97 – 1.05 | 0.81 |
| E/e’ | 1.00 | 0.98 – 1.02 | 0.71 | 1.00 | 0.99 – 1.01 | 0.85 |
| Interventricular septum (mm) | 1.02 | 0.98 – 1.06 | 0.34 | 1.01 | 0.99 – 1.03 | 0.40 |
| Posterior wall (mm) | 1.00 | 0.97 – 1.03 | 0.90 | 0.99 | 0.86 – 1.14 | 0.88 |
| Medications |  |  |  |  |  |  |
| Beta-blocker | 0.63 | 0.24 – 1.67 | 0.35 | 1.48 | 0.78 – 2.79 | 0.23 |
| RAS antagonist | 0.80 | 0.28 – 2.32 | 0.68 | 1.51 | 0.78 – 2.91 | 0.22 |
| MRA | 1.43 | 0.54 – 3.80 | 0.48 | 0.60 | 0.30 – 1.20 | 0.15 |
| Diuretic | 0.64 | 0.34 – 1.18 | 0.16 | 0.68 | 0.37 – 1.25 | 0.21 |
| Antiarrhythmic drugs | 0.59 | 0.18 – 1.91 | 0.34 | 0.63 | 0.20 – 1.96 | 0.42 |
| H_2_FPEF score | 0.88 | 0.61 – 1.24 | 0.46 | 0.87 | 0.65 – 1.12 | 0.35 |
| HFA-PEFF score | 0.88 | 0.67 – 1.14 | 0.33 | 0.85 | 0.64 – 1.13 | 0.28 |

csHR, cause-specific hazard ratio; sHR, subdistribution hazard ratio; CI, confidence interval. Other abbreviations are as in Table 1.
